# Characteristics of Stress Sensitivity in Heroin Use Disorder Patients during Their Opioid Agonist Treatment

**DOI:** 10.3390/ijerph20054566

**Published:** 2023-03-04

**Authors:** Filippo Della Rocca, Angelo G. I. Maremmani, Silvia Bacciardi, Matteo Pacini, Francesco Lamanna, Beniamino Tripodi, Mario Miccoli, Icro Maremmani

**Affiliations:** 1 Addiction Research Methods Institute, World Federation for the Treatment of Opioid Dependence, 225 Varick Street, Suite 402, New York, NY 10014, USA; 2 Department of Psychiatry and Addictions, Section of Psychiatry, North-Western Tuscany Local Health Unit, Tuscany NHS, Versilia Zone, Via Aurelia 335, 55041 Viareggio, Italy; 3 V.P. Dole Research Group, G. De Lisio Institute of Behavioural Sciences, Via di Pratale 3, 56121 Pisa, Italy; 4 Department of Psychiatry and Addictions, Section of Addictions, North-Western Tuscany Local Health Unit, Tuscany NHS, Pisa Zone, Via delle Torri 160, 56124 Pisa, Italy; 5 Department of Mental Health and Addictions, Division of Psychiatry, ASST Crema, Via Largo Dossena 2, 26013 Crema, Italy; 6 Department of Clinical and Experimental Medicine, University of Pisa, Via Roma 67, 56100 Pisa, Italy; 7 Saint Camillus International University of Health Sciences (UniCamillus), Via di Sant’Alessandro 8, 00131 Rome, Italy

**Keywords:** stress sensitivity, heroin addiction, OAT, heroin addiction history, psychopathology, behavioural covariates of heroin, cocaine and cannabis craving, subjective wellness

## Abstract

In the present study, performed on a sample of Heroin Use Disorder (HUD) patients undergoing Opioid Agonist Treatment (OAT), we attempted to explore the relationships between stress sensitivity and heroin addiction-related clinical aspects. HUD patients’ stress sensitivity was evaluated with the Heroin/PTSD-Spectrum questionnaire (H/PSTD-S). The Drug Addiction History Questionnaire (DAH-Q), the Symptomatological Check List-90 (SCL-90), and The Behavioural Covariate of Heroin Craving inventory (CRAV-HERO) were all used, as were the Deltito Subjective Wellness Scale (D-SWS), a self-report scale evaluating subjective well-being; the Cocaine Problem Severity Index (CPSI), a questionnaire determining the extent of a cocaine problem; and the Marijuana Craving Questionnaire (MC-Q), an instrument assessing craving for cannabinoids. We checked correlations between stress sensitivity and the extent of HUD clinical features and compared patients with and without problematic stress sensitivity. H/PTSD-S was positively correlated with patients’ income, altered mental status, legal problems, the lifetime different treatments index, the current treatment load index, and all SCL-90 indexes and factors. Regarding subjective well-being, stress sensitivity negatively correlated with the contrast best week (last five years) index. Patients with high-stress sensitivity were females with a low income. They exhibited a more severe mental status at treatment entry, greater difficulty in working adaptation, and legal problems during treatment. Additionally, these patients showed a higher level of psychopathology, more impairment in well-being, and more risky behaviours during treatment. Stress sensitivity, as H/PTSD-S, must be considered an outcome of HUD. HUD’s addiction history and clinical features are significant risk factors for H/PTSD-S. Therefore, social and behavioural impairment in HUD patients could be considered the clinical expression of the H/PTSD spectrum. In summary, the long-term outcome of HUD is not represented by drug-taking behaviours. Rather, the inability to cope with the contingent environmental conditions is the key feature of such a disorder. H/PTSD-S, therefore, should be seen as a syndrome caused by an acquired inability (increased salience) concerning regular (daily) life events.

## 1. Introduction

### 1.1. Towards a Patient-Tailored Therapy in Opioid Use Disorder

Substantial compelling evidence demonstrates that substance use per se is still a worldwide issue threatening individuals and communities. In vulnerable people, continuous use of rewarding substances can enduringly modify brain functions. The reward circuit’s responsiveness to reward and motivation, which are not drug-related, is decreased; sensitivity of the emotional circuits to stress is enhanced; and self-regulation functions are impaired. Such modifications may, in turn, induce dysfunctional behavioural changes, such as dysfunctional loss of control in drug seeking and drug use. Moreover, these brain changes can persist throughout an individual’s life. As a result, craving—the intense, urgent, and spontaneous desire for the rewarding substance—and relapse can last decades after clinical remission and abstinence [1,2]. In this context, addiction must be considered the end stage of such a pathological process. Namely, addiction is a chronic and relapsing brain disease characterised by impairing drug-induced neural modifications [3,4], nowadays widely recognised as a chronic and relapsing brain disease. On a clinical background, it has been observed that Heroin Use Disorder (HUD) patients, during long-term treatment, tend to show an impaired capacity to experience pleasure—anhedonia—and a more severe stress reaction to life events that may interfere with the rehabilitative program [5,6]. From a therapeutic point of view, managing addiction—particularly (HUD)—might be challenging for clinicians, as it implies extensive knowledge in managing long-term opioid medications and rehabilitation programs [7]. Opioid Agonist Treatment (OAT) has proved to be an effective intervention for HUD patients, as it allows a more effective reduction in heroin use than treatments that do not involve opioid medications [8]. However, neither biological nor clinical correlates have been standardised for treatment monitoring and outcome during OAT, so clinicians still set treatment strategies according to ‘good clinical practice’ (considering, for instance, clinical presentation, comorbidities, and urinalyses).

Moreover, retention in treatment is still the primary goal of OAT. So far, harm reduction is the most applied treatment strategy in dealing with HUD in professional medical treatment services worldwide [9,10]. On this clinical background, information from patients’ specific psychopathology, craving behavioural covariates, stress sensitivity, and individuals’ subjective well-being pointed to novel clinical information monitoring patients under OAT. It might be the key to better personalising diagnostic and therapeutic interventions, setting the basis for moving from harm reduction to patient-tailored therapy in OAT.

### 1.2. Psychopathology Specific to Substance Use Disorder

The diagnosis of Substance Use Disorder (SUD) is nowadays based on the presence of specifically identified behavioural symptoms [11]. Unlike most mental disorders, psychopathological syndromes occurring in SUD patients do not find space within the diagnostic criteria of SUD [11]. In parallel, any psychopathological signs and symptoms occurring in such patients have historically been confined to the “comorbidity” framework, as if psychopathology belonged exclusively to comorbid psychiatric disorders or underlying personality traits and not SUD [12,13,14,15].

Nevertheless, increasing scientific evidence has highlighted the clinical inadequacy of the diagnostic model proposed by the current reference manuals. In particular, recent epidemiological, genetic, neurobiological, and neuropsychological findings do not appear to correctly support DSM categories and definitions [3,16,17,18]. Therefore, many investigators have called for greater integration between such findings and the different diagnostic criteria of psychiatric illness. Specifically, it has been proposed that the psychopathological syndromes observed in psychiatric patients refer directly to the neurobiological substrates of behavioural patterns, including the addictive behaviours of SUD individuals [17,18]. In this context, it has been widely observed that psychopathological signs and symptoms—such as novelty seeking, irritability, restlessness, impulsivity, diminished interest in activities, dysphoria, boredom, depression, and attention and concentration difficulties—usually accompany addictive behaviours, thus contributing to the complete picture of the psychopathological profile of SUD patients and the persistence of their substance use [5,6,19,20].

Under those circumstances, in recent years, some Italian studies—mainly performed by the V.P. Dole research group at the Santa Chiara University Hospital in Pisa, Italy—have advanced the main idea that all psychiatric symptoms and clusters displayed in individuals with SUD should not be evaluated merely as manifestations of a generic psychiatric “comorbidity” [20]. Moreover, they further suggested the idea that such psychopathological symptoms—which mainly belong to the domains of anxiety, mood, and impulse control [19]—should instead be attributed directly, and by their very nature, to the addiction process, thus being a core clinical manifestation of SUD itself. Consequently, by advancing the hypothesis that addiction may have a specific psychopathology, such investigations have also explored some possible clinical implications, clarifying how information on patients’ psychopathological symptoms could be helpful in treatment choice [21] or outcome [22].

In particular, using an exploratory principal component factor analysis of the Self-Report Symptom Inventory (SCL-90) questionnaire—which is a self-report questionnaire used to measure psychopathological symptoms [23], widely used in the field of substance use [24,25,26]—a five-factor solution was studied in a sample of more than 2500 HUD individuals at treatment entry. These analyses led to the discovery of the five main domains: (1) the “worthlessness/being trapped (W/BT)” dimension that assembles obsessive-compulsive, depressive, and psychotic symptoms; (2) “somatic symptoms (SS)”, which is characterised by several somatic and anxious features, and resembles opioid withdrawal; (3) “sensitivity-psychoticism (S/P)” features, such as psychoticism and sensitivity; (4) “panic-anxiety (PA)”, which can be described as a fear of travelling by train or bus, going around alone, sensations of dizziness or fear of feeling sick, and the experience of acute anxiety; and (5) “violence-suicide (V/S)”, comprising aggressiveness against others and self-directed aggressiveness, with anger, rage, and breaking things up being fundamental elements of this domain. These five psychopathological syndromes are stable, specific to SUD [27,28,29,30,31,32,33,34,35,36,37,38,39,40,41,42,43,44,45,46,47,48], and are useful clinical features during the OAT [21,22].

### 1.3. Stress Sensitivity

The role of stress in addiction processes has been studied for decades. Many of the leading theories of addiction converge in recognised stress as a key factor in increasing vulnerability to addiction. On the one hand, several theoretical approaches emerging from psychological research consider substance use behaviour as a coping strategy to deal with stress—i.e., blunting inner tension, self-medicating, and reducing withdrawal-related distress. Over time, many coping paradigms have attempted to describe how stress is involved in substance use initiation, ongoing addiction processes, and relapse [49,50,51]. To explain, according to Chaplin’s ‘two-pathway model’ of substance use development, adolescents with a history of dysregulated stress reactivity may cope with increased environmental and internal stressors by using substances to either down-regulate overly high-stress reactivity or to up-regulate blunted stress reactivity [52]. This fact is consistent with the self-medication hypothesis of addiction [53] and stress-coping theories of adolescent substance use [54].

On the other side, stress theories of addiction—based on neurobiological models—have been proposed to explain how neuroadaptations in reward, learning, and stress pathways may enhance the key features of the addiction process, such as craving, impaired executive functions, and the inability to stop recurrent substance seeking behaviour and use despite adverse consequences [55,56].

Regardless of the SCL-90 psychopathological dimensions, stress sensitivity appears to be a cross-sectional key element in the psychic structure of SUD patients [55,57,58,59,60,61,62,63,64,65,66,67]. In general, the relationships between stress sensitivity and mental disorders have mainly been studied in recent years, both on biological and clinical grounds. However, much less is known about the relationship between stress sensitivity and SUD.

In summary, research on the relationship between stress and addiction mainly focused on stress sensitivity and its role in the developmental psychopathology of adolescent substance use and addiction. This fact is further true considering the known clinical implications of stress-related disorders—such as Post-Traumatic Stress Disorder (PTSD)—on the development of SUD [55,68,69].

#### 1.3.1. SUD as a Risk Factor in Increasing Individual Susceptibility to PTSD

Many neurobiological studies showed how substance use and its related acute and chronic behaviours could profoundly affect stress regulation. From a regular perspective, substance use increases stress response, even in individuals who do not exhibit comorbid conditions [60]. Furthermore, studies on the activity of the Hypothalamic-Pituitary-Adrenal (HPA) axis—which is widely considered a fundamental player in response to potential and actual stressors, controlling sympathetic, hormonal, and behavioural responses to stress [70]—revealed that baseline activity of the HPA-axis, specifically plasma ACTH and cortisol levels, is increased in individuals with SUD compared to healthy controls [25,71,72]. These findings suggest that SUD patients are even more vulnerable to the effects of stress, thus suggesting a higher stress sensitivity in such individuals than in non-addicted ones.

As stated above, much of the work exploring the relationship between substance use and stress has focused on the influence of chronic stress and trauma exposure on the likelihood of an individual developing SUD [55,68,69]. Nevertheless, we acknowledge that substance use itself may alter individuals’ stress sensitivity, changing how stress sensitivity contributes to the further development of SUDs over time [52,60]. Accordingly, a substance-abusing lifestyle might predispose substance users to experience stressful or traumatic events [68,73,74]. Moreover, data are available in the literature showing that drug-addicted subjects become more sensitive to stress the longer their history of drug addiction [55,62,75,76,77,78,79]. Along with the observation that stress sensitivity appears to be a cross-sectional key element in the psychic structure of SUD patients [55,57,58,59,60,61,62,63,64,65,66,67], in the present paper, we have attempted to address the topic of researching the relationship between stress and substance use from a different perspective. Specifically, the onset of substance use and alcohol misuse may precede the development of stress-related disorders—namely, the entire PTSD syndrome—in the clinical history, possibly contributing to increased individual susceptibility to them. In this case, a history of adverse and stressful experiences could have primarily induced substance use behaviours. In contrast, the neurobiological derangement caused by stress and psychotropic substances would be later responsible for the development of PTSD.

#### 1.3.2. Assessment of PTSD Spectrum in HUD Patients: Developing an Instrument to Evaluate the PTSD Spectrum

Several epidemiological data show frequent associations between PTSD and SUD. With this regard, more than 30% of patients suffering from SUD were found to meet the criteria for current PTSD, and half of them for lifetime PTSD [55,68,69]. Moreover, the co-occurrence of SUD-PTSD is associated with more severe psychopathology, more severe addiction history, worse executive functions, higher rates of overdoses, attempted suicide, and poorer treatment outcomes than those with PTSD or SUD alone [80,81,82]. Therefore, it appears clear that adequately screening for PTSD might be a crucial step in creating a patient-tailored therapy for SUD patients. Thus, assessing PTSD features must be considered clinically required during a daily routine. The need to develop a reliable tool to evaluate PTSD characteristics in SUD patients led to a first conceptual problem. In this regard, it is worth mentioning that an undefined area of the interactions between stress, traumatic experiences, and substance use was observed in many of these cases without any possibility of recognising a well-defined sequence in the clinical history and related neurobiological changes. Many patients also do not fulfil the Diagnostic and Statistical Manual of Mental Disorders (DSM) criteria for PTSD diagnosis, even though they show clear evidence of stressful and traumatic implications in developing substance use behaviours.

Moving from the role mentioned above of stress sensitivity in mediating the relationship between SUD and PTSD, the research group V.P. Dole of the Santa Chiara University Hospital of Pisa, Italy, has attempted, in recent years, to address this issue through the study of stress-reactivity indices in patients with HUD. They came to conceptualise a ‘PTSD-Spectrum’, based upon a concept that uses individual DSM criterion symptoms as a starting point and extends the DSM description to encompass the halo of surrounding clinical phenomena—following a similar conceptualisation for other psychiatric disorders, such as Bipolar Disorder [83]. These include associated features described in the DSM as well as symptoms, maladaptive behavioural traits, and temperamental characteristics that do not appear in the DSM. However, such features seemed to be based on real-world settings, providing the diagnostic categories with a useful and more complete characterisation of psychopathological dimensions.

To assess the PTSD-Spectrum in patients with HUD, the V.P. Dole research group developed the ‘Reaction to loss and traumatic life events—Heroin/PTSD-Spectrum’ (H/PTSD-S) inventory) [84,85], derived from the ‘Trauma and Loss Spectrum-Self-Report, Instrument Questionnaire, Lifetime version’ (TALS-SR) [86,87]. Such an instrument showed the ability to adequately explore the lifetime experience of a range of loss or traumatic events comprising lifetime symptoms, behaviours, and personal characteristics, which might represent manifestations and risk factors for the development of a stress response syndrome, thus allowing the study of patients’ stress sensitivity.

The aims were to continue the process of validation of the H/PTSD-S inventory [84,88], and, in the present study, we assessed the correlations between the H/PTSD-S inventory with heroin addiction-related clinical aspects (addiction history, comorbid substance use, severity of addictive behaviours, and subjective wellness) and the severity of the psychopathological symptoms. In this way, it is possible to define HUD patients’ stress sensitivity better.

## 2. Materials and Methods

### 2.1. Design of the Study

The data were collected using the HUDPyscho-study, an ongoing cross-sectional cohort study from Pisa, Italy’s Drug Addiction Service (SerD). This study is a naturalistic, single-centre, observational, and non-interventional cohort study applying the usual procedure consistent with daily clinical practice. This study allows researchers to examine clinical and psychopathological features, stress sensitivity, and the quality of life in HUD patients during the OAT through a single administration of questionnaires during treatment. The University of Pisa Ethical Committee has previously approved the HUDPyscho-study (ID 22656, CEAVNO 21.07.2022).

### 2.2. Sample

The study’s participants were recruited from the Drug Addiction Service (SerD) in Pisa, Italy. It is a local public unit for drug addiction located in the north-western part of central Italy (province of Pisa, Tuscany region). Persons referred to the service were mainly residing in the same area. We did not use specific criteria for eligibility other than the “wish to be treated” and “wanting to participate” in the survey.

Patients who were at least 18 years and diagnosed with Opioid Use Disorder (OUD)—according to the DSM-5 criteria [11]—and in treatment with agonists of opioid medications were included in the study. Patients who could not provide written informed consent to study participation were excluded. The clinical assessment of each participant has been performed through a single administration of questionnaires (around 2 h), both in a self-report and in a clinician-report modality. Both modalities were temporarily synchronised so that all clinical data could be considered a function of the patient’s time in OAT.

Data were obtained on all patients who voluntarily agreed to be enrolled in the present study at any time between June 2022 and October 2022; to avoid discrepancies, they were, without exception, evaluated by the same physician on duty (FDR).

In the Drug Addiction service of Pisa, an OAT, according to the Dole and Nyswander (D&N) treatment methodology, has been used since its foundation [89,90].

46 HUD patients in OAT—referring to the Drug Addiction service of Pisa—were asked to participate in the present study. Two patients declined to be enrolled: one was suspicious, possibly due to psychotic symptoms, and one did not explain any reason. The whole enrolled sample consisted of 44 patients, with a mean age of 42.14 ± 11.0 years (18–62 ranging), and 33 (75.0%) patients were males while 11 (25.0%) were females.

### 2.3. Instruments

The assessment of patients was achieved with the following questionnaires:

- Drug Addiction History Questionnaire (DAH-Q) for the standard demographic and drug history data collection.
- Symptomatological Check List-90 (SCL-90) is a list of psychopathology symptoms.
- Heroin Craving Behavioural covariate (CRAV–HERO) is an inventory of heroin addictive behaviour.
- Heroin/PTSD-Spectrum questionnaire (H/PSTD-S), a new instrument developed to evaluate stress-sensitivity in HUD patients.
- Deltito Subjective Wellness Scale (D-SWS) is a self-report measure evaluating the quality of life.
- Cocaine Problem Severity Index (CPSI) is a questionnaire determining the extent of a cocaine problem.
- The Marijuana Craving Questionnaire (MC-Q) assesses the craving for cannabinoids.

#### 2.3.1. Drug Addiction History Questionnaire (DAH-Q) 

The DAH-Q is a comprehensive questionnaire regarding heroin addiction history [91]. It takes the form of a semi-structured interview comprising a multidimensional questionnaire. Specifically, seven areas are enclosed: I–Somatic pathology (8 items, Cronbach’s alpha = 0.60); II–Mental status (12 items, Cronbach’s alpha = 0.78); III–Social adjustment (5 items, Cronbach’s alpha = 0.63); IV–Co-occurring substance use (7 items, Cronbach’s alpha = 0.72); V–Modality of use (5 items, Cronbach’s alpha = 0.53); VI–Past and Current Treatment History (8 items, Cronbach’s alpha = 0.61); and VII–Longitudinal addiction-history aspects (3 items, Cronbach’s alpha = 0.71). The III area is composed of (a) work, (b) family, (c) intimacy, (d) social/leisure, and (e) legal problems. The design of its related questionnaires is based on structures requiring dichotomous (presence/absence) answers. For each area, the score is reported as a ratio—based on the number of aspects present in that specific area and in relation to the maximum number of items listed for the same area. A ratio = 1 represents the presence of all investigated aspects referring to that area.

#### 2.3.2. Symptomatological Check List-90 (SCL-90)

According to the methodology of Maremmani et al. [92], the Symptomatological Check List-90 (SCL-90) is a self-report rating scale evaluating outpatients’ psychiatric and symptomatic behaviours. It consists of 90 items, with five levels of severity (ranging from a minimum of ‘Not at all’ to ‘Extremely severe’), evaluating psychopathological severity across nine dimensions, including internalising and externalising symptoms. It was initially developed by Derogatis et al. [23]. By including HUD for the first time, the 90 items have been rearranged by the V.P. Dole research group into five main dimensions, which are viewed as the background of a large majority of symptomatic behaviours observed in individuals with such a disorder. The primary symptomatologic dimensions are (1) Worthlessness/Being Trapped (W/BT), (2) Somatic Symptoms (SS), (3) Sensitivity/Psychoticism (S/P), (4) Panic Anxiety (PA), and (5) Violence/Suicide (V/S). These five main domains have been primarily validated in over 2500 substance-use disorder individuals. According to the highest Z-score for each factor, participating subjects were distributed into five samples (called “dominant groups”). The largest group of patients was the dominant group distinguished by somatic symptoms (24%). The second largest group was the ‘panic anxiety’ one (22%), followed by the violence/suicide group (20%), the sensitivity/psychoticism group (20%), and, lastly, the worthlessness/being trapped group (14%). Each of these five dimensions was independent of the others and showed no significant overlap [92]. Following this enquiry, several further studies were conducted to ascertain whether this psychopathological structure might be considered a stable trait or a variable state that potential confounding factors might condition [27,28,29,30,31,32,33,34,35,36,37,38,39,40,41,42,43,44,45,46,47,48]. Nowadays, the SCL-90 questionnaire might be regarded as a reliable instrument to assess psychopathology specific to SUD clinically.

#### 2.3.3. Heroin Craving Behavioural Covariate (CRAV-HERO)

The Heroin Craving Behavioural Covariate chart (CRAV-Hero) allows researchers to record both the presence and severity of addictive behaviours, investigating the craving of HUD individuals. Using focus groups and brainstorming methodology, an expert panel belonging to the V.P. Dole Research Group at the Dual Disorder Unit of the Santa Chiara University Hospital in Pisa, Italy, selected items to be included in the inventory. Focus groups were open to rehabilitated HUD patients. In these ways, the possibility that specific craving behaviours accurately reflected what they were intended to reflect was stressed so that the results could be applied to real-world settings (content or construct validity of the inventory). Thirteen behaviours were selected. For each of these, the five available answers were related to five different levels of severity. Out of each set of five solutions proposed for every question, only one could be picked; the answers were presented in an order corresponding to a stepwise increase in the level of craving. In this way, a score (from 0 to 4) was attributed to each question, and by adding up the results, the total score was in line with craving intensity. Because the overall damage done by craving should be estimated in terms of the total costs (going beyond a merely economic level) that a subject is willing to pay to obtain the drug, the indirect questions are focused on four main themes: (1) exchange, (2) time, (3) risk, and (4) cue-induced/environmental stimuli to use. The CRAV-HERO has been validated in Italian HUD patients [93].

#### 2.3.4. Deltito Subjective Wellness Scale (D-SWS)

The Deltito Subjective Wellness Scale (D-SWS) looks at three periods in assessing the patients’ well-being. Time one is defined as ‘now and characteristic of the past week’; time two is defined as ‘during the worst week of your current or most recent depressive episode—within the last year’; and time three is defined as ‘the best week you have had in the previous five years’. The D-SWS is intended to be directly filled out by the patient, following an explanation by the clinician to verify whether the patient understands. It explores patients’ self-rated quality of life, including, among others, hobbies, sexual life, and occupational features. Each HUD participant can choose among ‘0’ (No Way Whatsoever), ‘1’ (To a Minor Extent), ‘2’ (To a Major Extent), and ‘3’ (To the Highest Extent Possible). D-SWS evaluates the patient’s quality of life based on their subjective feelings, so it is free from clinical judgments. The assessment takes place through a temporal dimension, providing information about the disorder’s progression over time, the subjective functional impact on everyday life and the treatment outcome.

#### 2.3.5. Cocaine Problem Severity Index (CPSI)

The Cocaine Problem Severity Index (CPSI) may help determine the extent of a cocaine problem. The CPSI was initially developed by Rawson et al. in 1989 [94] for the clinical assessment of cocaine use. Using the CPSI questionnaire, it is possible to assign each cocaine user to a four-subgroup set, namely ‘experimental/recreational use’, ‘cocaine abuse with a significant problem’, ‘cocaine dependence requiring assistance’, and ‘severe dependence’. Even though they might be using cocaine only in a social context, patients showing an ‘experimental/recreational use’ (E/R) are prone to increase their use if the stress increases or the substance becomes more accessible. In patients belonging to the ‘cocaine abuse with a significant problem’ (CASP) group, the effects of cocaine on their life tend to be substantial. These patients are recommended to obtain a professional evaluation to help them determine the most effective way to deal with problems related to cocaine use. In patients with ‘cocaine dependence requiring assistance’ (CDRA), cocaine use is a severe problem, and they need to seek assistance to learn what addiction is and how to deal with it. Lastly, it might be difficult for patients in the ‘severe dependence’ (SD) group to regain control of their life without hospitalisation. The CPSI questionnaire contained 18 items, each requiring multiple answers corresponding to various levels of severity from 0 to 8; thus, the maximum craving score was 86 [94].

#### 2.3.6. Marijuana Craving Questionnaire (MC-Q)

The Marijuana Craving Questionnaire (MCQ) is a multidimensional questionnaire developed by Heishman et al. to assess marijuana cravings [95]. The development of the MCQ was based on the model of the ‘Questionnaire on Smoking Urges’ [96] and the ‘Cocaine Craving Questionnaire’ [97]. Items on the MCQ were drawn from five theoretical conceptualisations of craving. The proposed five categories were: (1) desire to use marijuana, (2) anticipation of positive outcomes from marijuana use, (3) anticipation of relief from withdrawal symptoms or negative mood, (4) intention and planning to use marijuana, and (5) lack of control over marijuana use.

#### 2.3.7. Stress Sensitivity—Heroin/PTSD-Spectrum (H/PTSD-S)

The H/PTSD-S inventory has been previously proposed to explore the PTSD spectrum in HUD patients. It is a short self-report questionnaire comprising the 30 most significant items retained from the TALS-SR form. To assess the PTSD spectrum, the V.P. Dole research group used the ‘Trauma and Loss Spectrum-Self-Report, Instrument Questionnaire, Lifetime version’ (TALS-SR) [86,87]. TALS-SR includes 116 items exploring the lifetime experience of a range of loss or traumatic events comprising lifetime symptoms, behaviours, and personal characteristics that might represent manifestations and risk factors for developing a stress response syndrome. The validity of TALS-SR is well established [86,87,98], but some limitations in using it with HUD patients should be mentioned. Specifically, when addicted patients approach treatment settings, they typically make a spontaneous request for help, revealing a different motivational status and a constant ambivalence towards compliance with treatment, which they may be aware of to a certain degree. Their willingness to be clinically assessed before starting treatment is very low.

Moreover, their ability to adequately maintain their attention to fill in a rating scale is minimal at treatment entry—and during the various treatment phases. So, the time allocated to rating scale evaluation must be limited. The TALS-SR questionnaire needs a consistent amount of time to be completed. Thus, a shorter TALS-SR form was required to assess HUD patients. Items from TALS were selected to obtain a reduced form for HUD patients, making it possible to differentiate patients with and without a PTSD spectrum comparable with the one developed by the survivors of the L’Aquila (Italy) 2009 earthquake [98]. Eventually, the V.P. Dole research group developed the ‘Reaction to loss and traumatic life events—Heroin/PTSD-Spectrum’ (H/PTSD-S) inventory, which consists of 30 dichotomic items. The number of items in the TALS questionnaire was reduced using V.P. Dole’s TALS database, in which 235 questionnaires previously administered to a sample of ‘typical respondents’—evaluated on entering or during an OAT in previous research protocols were stored. Patients’ questionnaires were divided into two groups, indicating or not indicating the presence of H/PTSD-S, according to the methodology described in Dell’Osso et al. [57]. Logistic regression analysis for each TALS domain was then performed, using the H/PTSD-S presence as a criterion and domain items as predictors.

Only cases with *p* < 0.05 and OR above 3.0 were retained. ROC analysis determined a cut-off value and distinguished the H/PTSD-S presence/absence. The cut-off’s acceptable percentage of sensitivity and specificity was set to 80%. The confidence level of the Area Under the Curve (AUC) was 95%, and AUC was considered statistically significant with a *p*-value < 0.05. It was assumed that the 25–75% interval would be adequate in testing the discriminative effect of the selected items in our reduced TALS inventory, as it would minimise the floor effect. Internal consistency (reliability) was estimated by applying Cronbach’s alpha test. This type of spectrum was renamed Heroin/PTSD-Spectrum (H/PSTD-S) [84,85]. The cut-off value determined by the ROC analysis was 11. All the items demonstrated adequate variability. The internal consistency (reliability) estimated using Cronbach’s alpha was optimal (0.88). The proposed H/PTSD-S inventory, founded on achieving satisfactory internal consistency, measures the stress reactivity construct.

### 2.4. Data Analysis

The Shapiro–Wilk test was performed to verify the normality of distributions. Spearman’s correlation coefficient analysed correlations that concern quantitative and ordinal variables. Comparisons between groups were analysed using Fischer’s exact test for categorical variables and the Mann–Whitney U test for ordinal variables. Missing data were excluded from the analyses. As this is an exploratory study, statistical significance was considered for *p* ≤ 0.05.

## 3. Results

### 3.1. Demographic and Clinical Characteristics

Most of the participants were single (68.2%), highly educated—(>eight years of education) (52.3%), unemployed (55.3%), from a white-collar parental family (62.2%), receiving adequate income (75.0%), and living alone (55.8%). The number of patients who reported no lifetime somatic pathology was 15%. On average, the sample presented 2.24/8 lifetime somatic pathologies and 3.84/12 aspects of altered mental status. Regarding the current social adjustment, most of the participants were satisfied with their household situation (53.3%), intimacy (57.7%), and social/leisure activity (63.6%). Furthermore, most of them had no legal problems (54.8%). Regarding lifetime cooccurring substance use—other than heroin—the lifetime use of CNS-Depressants was reported in 73.3% of cases, the use of CNS-Stimulants in 82.9%, the use of Hallucinogens in 37.0%, the problematic use of alcohol in 59.4%, the use of cannabis in 88.6%, and, eventually, the use of inhalants in 4.0%. On average, the sample presented 4.20/6 cooccurring substances. Regarding the modality of heroin use, the sample was characterised by a ‘multiday’ heroin intake modality (90.9%) and an unstable modality of use—they used to lead an existence that is not likely to be accepted to social conventions (they are considered either ‘fanatic’, ‘loners’, or ‘violent’) (74.2%). Moreover, most participants were in stage 3 of their heroin use disorder, the ‘revolving door’ stage (96.8%), and reported a clinical typology related to psycho-social antecedents—that is, these patients are mostly highly responsive to personal and environmental stimuli or issues (91.2%). On average, the sample presented 4.95/12-lifetime different treatments and 2.76/4 different current treatments. On the whole, the mean problematic areas load was 4.4/10.

The age of first contact with the substance of primary use—heroin—was 20.71 ± 5.9 years; the age of continuous use was 28.78 ± 9.4 years; andthe dependence length was 152.75 ± 108.5 months. The age at the first treatment was 30.93 ± 8.6 years, and the current treatment length was 27.54 ± 44.0 months. The time between the age at first contact and continuous use was 6.54 ± 8.5 years; between first use and first treatment was 9.07 ± 8.5 years; and between age at ongoing use and first treatment was 2.48 ± 3.0 years.

Furthermore, participants were more likely to be diagnosed with Bipolar Spectrum (29.5%) than with Recurrent Depression (6.8%), Anxiety Disorders (4.5%), and Chronic Psychosis (2.3%). Most were not reported to display a Dual Disorder (56.8%).

Eventually, 63.6% of patients were undergoing a Methadone Maintenance Treatment (with a mean dosage of 81.96 ± 42.1 mg/day), 15.9% were on a Buprenorphine Maintenance Treatment (11.14 ± 7.5 mg/day), and 20.5% with other medications.

Most of the sample reported the presence of a Heroin PTSD Spectrum (N = 34, 77.3%).

### 3.2. Bivariate Correlation between the Severity of Stress Sensitivity, Length of Current Treatment, and Other Clinical Aspects

Table 1 shows significant correlations between stress sensitivity, current treatment duration (months), and other clinical aspects. The Total PTSD Spectrum Score represents stress sensitivity.

Regarding demographic and drug addiction history data, stress sensitivity positively correlated with participants’ income, altered mental status, legal problems, lifetime different treatments index, and current treatment load index.

Regarding the SCL-90 psychopathological indices, stress sensitivity showed a positive correlation with each of the variables, namely, with the global score index, the positive symptom index, the total SCL score, the positive symptom distress (severity), and the severity of each SCL-90 factor—W/BT, SS, S/P, PA and V/S dimension.

Regarding indices of quality of life retrieved from the D-SWS, the severity of stress sensitivity showed a high negative correlation with the contrast best week (last year) index.

No correlation was found between stress sensitivity and behavioural covariates of heroin (rho = 0.25; *p* = 103) and cocaine (rho = 0.26; *p* = 0.087) and between stress sensitivity and cannabis craving (rho = −0.04; *p* = 0.783).

### 3.3. Differences between Patients with and without Heroin/Post-TrauPosttraumaticisorder—Spectrum (H/PTSD-S)

Table 2 shows demographic and clinical differences between patients with (N = 34) and without (N = 10) Heroin PTSD-Spectrum.

Concerning demographic data, the two groups differed in gender and income. Specifically, patients without H/PTSD-S were all males and with adequate income. No significant differences were found regarding age (U = 155.50; z = −0.40; *p* = 0.689), marital status (*p* = 1.000), education (*p* = 1.000), occupation (χ^2^ = 1.88; *p* = 0.389), parental family working activity (χ^2^ = 1.85; *p* = 0.396), and living situation (*p* = 1.000).

Regarding the heroin addiction history, a significant difference was observed between the two groups regarding the problematic area ‘Mental Status’, with the group with H/PTSD-S reporting a higher score than the group without H/PTSD-S. Another difference was observed regarding the presence of an occupation, which was higher in patients without H/PTSD-S, and also in legal problems, which were higher in patients with H/PTSD-S.

No differences were observed regarding heroin intake (*p* = 0.523), modality of use (*p* = 0.335), HUD stages (*p* = 1.00), clinical typology (*p* = 1.00), diagnoses (χ^2^ = 8.65; *p* = 0.070), presence of dual disorder (*p* = 0.474), use of CNS depressants (*p* = 0.060), CNS stimulants (*p* = 0.117), hallucinogens (*p* = 0.666), age at heroin first contact (U = 88.00; z = 0.19; *p* = 0.872), age at heroin continuous use (U = 69.50; z = 0.91; *p* = 0.377), dependence length (U = 43.50; z = −0.84; *p* = 0.413), age at first treatment (U = 82.00; z = 0.51; *p* = 0.631), present treatment length (U = 134.50; z = 1.04; *p* = 0.304), the time between age at first contact and age at continuous use latency (U = 55.00; z = 0.16; *p* = 0.900), the time between age at first use and age at first treatment latency (U = 81.50; z = 0.87; *p* = 0.395), the time between age at continuous use and age at first treatment latency (U = 38.00; z = 0.74; *p* = 0.514), other problematic areas (somatic complications (U = 87.00; z = −0.39; *p* = 0.717), substance use (U = 68.50; z = −1.57; *p* = 0.122), lifetime different treatments (U = 61.50; z = −1.73; *p* = 0.084), current treatment load (U = 72.00; z = −1.59; *p* = 0.166), and the total problematic areas score (U = 71.00; z = −1.45; *p* = 0.154).

Regarding the SCL-90 psychopathological indices, the two groups showed significant differences concerning the global score index, the positive symptom index score, and the positive symptoms distress. The two groups also differed concerning W/BT, SS, SP, and V/S psychopathological dimensions. No difference was found in comparing the five prominent psychopathological typologies (χ^2^ = 4.65; *p* = 0.324). In all these variables, patients with H/PTSD-S reported higher scores.

Regarding the D-SWS indices, a highly significant difference between the two groups was found regarding the ‘contrast’ with the poor week in the last year and the ‘contrast’ with the best week in the previous five years. Specifically, patients without H/PTSD-S reported much improved subjective well-being compared to the previous year’s poor week and the ‘contrast’ with the best week in the last five years.

Eventually, no significant difference was observed between groups regarding the heroin addictive behaviour covariate—CRAV-ERO total score (U = 122.50; z = −1.61; *p* = 0.186)—cocaine problem severity index (U = 113.50; z = −1.60; *p* = 0.141), and cannabis craving severity (U = 214.00; z = 1.52; *p* = 0.227).

## 4. Discussion

### 4.1. Demographic and Clinical Characteristics

The sample in the present study was predominantly male, and most participants reported being single, highly educated, and unemployed, yet overall they reported adequate income and living alone. These demographic characteristics are typical of the Italian drug addict population, where poverty is relatively rare. Furthermore, most participants in the present study did not report any legal problems. Regardless of the treatment duration and what might be expected for a sample retrieved from a real-world setting, this demographic characteristic is consistent with the literature regarding expectations of excellent clinical practice during OAT. Therefore, this high percentage of patients not reporting legal problems should be interpreted as a virtuous consequence of OAT. To explain, according to the current literature, the main objectives of long-term management of patients suffering from Opioid Use Disorder (OUD) include the reduction of the risk of death and disease—a basic goal, which is better known as ‘harm reduction’—improved mental health and outlook, and restoration of compromised social role due to issues such as unemployment, disrupted family relationships, and involvement with the criminal justice system [99,100].

Nevertheless, a very high percentage of participants reported an actual history of ‘addiction’ (i.e., having experienced a ‘revolving door’ life or repeated detoxifications). On the one hand, the high proportion of patients classified as stage 3 in their natural history of HUD at treatment entry should not be surprising, as HUD patients are more likely to get in treatment—and to retain in treatment—in a later stage in the course of their disorder than in any earlier stage (i.e., the ‘honeymoon’ stage and the intermediate or ‘dose-increasing’ stage [101]. A “revolving door” situation is characterised by a dramatic, unfolding sequence of events, such as being treated, dropping out of treatment, having an argument, being arrested, being admitted to the hospital, returning to treatment, and so on. This phase is of fundamental clinical importance, as the risk of death from “overdose” is higher than in the previous steps. This fact is due to detoxification, which should be viewed as a gradual decline in opioid tolerance.

Along with this, the onset of craving for the substance tends to push the subject to occasional heroin use. At this stage, taking a dose of heroin equal to the quantity taken during the’ tolerance period’ is at high risk of causing an “overdose” [102]. Furthermore, such a clinical characteristic should be considered a stable feature of the disorder, being more related to the patient’s drug addiction history than any current condition at their treatment entry, including ongoing treatment. Accordingly, in the present study, most participants had undergone several previous treatment programmes at the time of the evaluation—this also comes from data regarding participants’ mean age, age at the first treatment, and current treatment length.

On the other hand, the high rates of the less-than-daily modality of heroin intake among unstable users during treatment imply that most patients reduced but did not stop heroin use during treatment. This result does not conform to the Dole and Nyswander methodology, and its most likely interpretation is linked to a suboptimal result of the current treatment [103]. Regarding the rates of psychiatric diagnoses—Bipolar Spectrum, Recurrent Depression, Anxiety Disorders, Chronic Psychosis, and, on the whole, a Dual Disorder diagnosis (43.2%)—we can state that our sample is consistent with the literature, confirming the high occurrence of a bipolar spectrum among dual disorder patients [104,105,106,107,108]. Furthermore, our sample reported a lifetime co-occurring substance use of CNS-Stimulants, CNS-Depressants, and Hallucinogens [109,110,111,112].

Interestingly, an overall improvement was found when comparing the least satisfying week over a longer period (past five years) with the current week’s satisfaction. Considering the mean duration of the current treatment (27.54 months), it is likely that the findings described above represent three different stages of drug addiction history in our sample. Specifically, the participants’ worst week over five years must be related to a subjective condition before entry into treatment, thus representing typically unstable and impaired life situations in HUD patients during stage 3 of the disorder. To the best of our knowledge, this is the first time that a degree of subjective well-being contrasts has been assessed in a sample of HUD patients according to the Deltito–Subjective Wellness Scale.

### 4.2. Bivariate Correlation between the Severity of Stress Sensitivity and Other Clinical Aspects

The present study found a high positive correlation between the severity of stress sensitivity and many clinical aspects. In particular, participants’ income, altered mental status areas, legal problems, lifetime load of different treatments, and current treatment load correlated with the severity of the PTSD-spectrum. In a previous study performed by the V.P. Dole research group on HUD patients, the severity of symptoms related to the PTSD-spectrum seemed to be positively correlated with both the duration and the intensity of the stressful condition. Higher levels of PTSD-Spectrum symptomatology in subjects with a long history of heroin abuse were found [57,113]. Similar results were again observed in a study on long-term survivors of Hodgkin’s and non-Hodgkin’s lymphoma: subjects whose disease began at an earlier age suffered significantly more intense intrusion and avoidance symptoms [114].

The positive correlation between the general index of psychopathology and specific psychopathological syndromes of addictions is not surprising. In a previous study [35], after three months of stay in a TC (Therapeutic Community), a general reduction of SCL-90 severity was accompanied by a reduction in the frequency of those dimensions which were most closely related to the intoxication/withdrawal state and with active substance use-related behaviour (SS and W/BT). The least frequent variation concerns the patients allocated in the dimensions most involved in the addiction processes (PA and V/S). The present study underlines the close link between changes in stress sensitivity and psychopathology. Likewise, a negative correlation was found between stress sensitivity and the ‘contrast best week of the last five years’ at the DSWS, thus suggesting a trend to report a long-term worsening rate of subjective well-being in patients with greater stress sensitivity.

### 4.3. Differences between Patients with and without Heroin/Post-TrauPosttraumaticisorder—Spectrum (H/PTSD-S)

Regarding demographics, significant differences between patients with and without H/PTSD-S were found in sex and income distribution. These data might suggest a depressive dimension of H/PTSD-S, as depression is more prevalent in women. In addition, women are known to be at particular risk of developing PTSD [115]. A significant difference was also found in the ‘Mental Status’ problematic area, which was more impaired in patients with H/PTSD-S. Additionally, occupational and legal problems were more complicated in the H/PTSD-S group. Consistently, previous findings regarding stress sensitivity in unemployed and shift workers reported higher overall impairment, higher perceived stress, and lower subjective wellness while exhibiting elevated hair cortisol concentration [116].

As regards psychopathological indices and syndromes, the severity of such symptomatology was more significant in the H/PTSD-S group. Namely, patients with H/PTSD-S reported higher levels of severity in each of the five psychopathological dimensions. This result should not be surprising since stress sensitivity and psychopathology appear closely related. To explain, women who had experienced lifetime intimate partner violence were twice as likely to report depressive disorders, four times more likely to meet the criteria for an anxiety disorder, and seven times more likely to be diagnosed with PTSD [117]. Mental disorders increase the perpetuation of partner violence [118]. Indeed, the study of Van Reekum et al. showed that the occurrence of psychological symptoms increases the likelihood of interpersonal conflicts in both men and women [119].

Moreover, Mills et al. [82] described the impact of current and lifelong PTSD on long-term recovery from heroin dependence among participants who took part in the 11-year follow-up of the Australian Treatment Outcome Study (ATOS), a prospective naturalistic longitudinal study involving 615 people with heroin dependence recruited from Sydney, Australia, in 2001–2002. Seventy-one per cent of the cohort (n = 431) were re-interviewed 11-year later the study enrolment. Outcomes reviewed included heroin and other substance use, addiction, general physical and mental health, depression, PTSD, occupation, the incidence of trauma exposure, overdose, incarceration, and attempted suicide over the 11-year follow up. Despite having a poorer profile at baseline, individuals with current PTSD or a history of PTSD at baseline demonstrated similar levels of improvement as those without a history of PTSD at baseline in all outcome domains during the 11-year follow up. Nevertheless, PTSD was associated with consistently higher levels of major depression, attempted suicide, subsequent trauma exposure, and poorer occupational functioning across the 11-year follow up. These findings highlight the importance of occupational rehabilitation interventions, reducing the likelihood of re-traumatisation. Eventually, in the present study, no difference was found between the two groups as regards the prominence of the five different SCL-90 dimensions.

Our findings were further confirmed by the differences observed between the two groups in terms of subjective well-being, according to the D-SWS. Notably, both groups (patients with H/PTSD-S and patients without H/PTSD-S) reported two levels of improvement over time regarding subjective well-being. In particular, both groups reported improved subjective well-being when comparing the current week’s subjective well-being to last year’s poor week and the best week over five years. However, compared to patients without H/PTSD-S, patients with H/PTSD-S showed a lower rate of improvement on both the ‘contrast’ measures.

This report seems to suggest a different specific outcome in the context of the OAT. Patients with HUD—especially in the third stage of the disease—before entering treatment, actively seek a better quality of life and therefore require entry into treatment. The present study suggests that improvement in subjective well-being may be a key factor for the retention in treatment for such individuals after initiation of treatment. Therefore, beyond the behavioural, clinical, and psychopathological aspects, the subjective perception of the patients’ high being should be an essential factor during the OAT.

The attention of researchers and health professionals to the reported well-being of opioid users is growing [120,121,122]. OAT is known to improve the quality of life of patients. Nonetheless, measuring and standardising these outcomes has proven challenging as there is no consensus on which outcome measures of functioning or quality of life are related to drug use, which still generates much debate. The present study uses a triple scale of subjective well-being to easily compare the patient’s perception in three different periods to assess patients’ quality of life. Moreover, to the best of our knowledge, this is the first time that a degree of subjective well-being—and its contrasts—has been assessed in a sample of HUD patients according to the Deltito–Subjective Wellness Scale.

The limitations of this study include the number of examined patients is the major limitation of this study. In addition, running many analyses, a Type 1 error is possible. The group without H/PTSD-S was made up exclusively of males, and a poor sample size prevented multivariate analyses from being performed. Furthermore, much data in the present study were collected through self-report instruments, particularly concerning psychopathological, stress sensitivity, and patients’ well-being characteristics, thus influencing PTSD symptoms collection and diagnosis. A self-assessment of PTSD symptoms may be considered less accurate than the reports of the physicians. Moreover, other theories have been posited to explain the associations between stress on the one hand and substance abuse and addiction on the other, including tension reduction theory, stress-dampening response, and common genetic risk factors. Moreover, D-SWS has not been standardised yet, and CPSI and MC-Q have not been standardised in Italian.

We acknowledge that other theories have been posited to explain the associations between stress, on the one hand, and substance use behaviours and addiction, on the other, including stress-coping theories and common genetic risk factors. Furthermore, it was not the purpose of this study to explore the aetiology of PTSD in individuals with HUD. Instead, we aimed to describe the characteristics of PTSD-Spectrum. Further studies would better clarify the relationship between addiction and PTSD in terms of mutual impact.

## 5. Clinical and Research Implications

There is still no consensus on which perspective should be prioritised to reflect better treatment benefits: adverse social outcomes to avoid, symptom reduction, drug-taking behaviour, or patient perspective.

On this clinical background, information from patient-specific psychopathology, behavioural covariates of craving, stress sensitivity, and individuals’ subjective well-being could indicate new clinical information for monitoring patients undergoing OAT. It could be the key to better tailoring diagnostic and therapeutic interventions, laying the foundations for moving from harm reduction to patient-tailored therapy in OAT.

Specifically, the present study’s findings highlight the importance of assessing patients’ stress sensitivity—thus screening for PTSD—and subjective well-being during OAT. H/PTSD-S patients might display a worse clinical picture and outcome. Interventions targeting occupational rehabilitation, reducing the likelihood of re-traumatisation, and addressing PTSD and associated comorbidities should be considered.

## 6. Conclusions

In conclusion, in the present study, we suggested the presence of a PTSD-Spectrum occurring in a sample of HUD patients (H/PTSD-S). This syndrome was observed in a subsample of patients with high sensitivity to stress during OAT and showed a close correlation with the psychopathological syndromes of patients with HUD. Compared with patients without H/PTSD-S, patients with H/PTSD-S were mainly females with low income. They exhibited a more severe mental status at treatment entry, greater difficulty in working adjustment and legal problems during treatment. Additionally, H/PTSD-S patients showed a higher level of psychopathology and a lower improvement in subjective well-being during OAT.

Eventually, we further suggest that H/PTSD-Spectrum should be considered an outcome of HUD. Both addiction history and clinical features of HUD, in turn, seem to become significant risk factors for the onset of H/PTSD-S. Therefore, in such a perspective, social and behavioural impairment in HUD patients could be considered the clinical expression of the H/PTSD-spectrum. According to this view, the long-term outcome of HUD is not represented by drug-taking behaviours. Rather, the inability to cope with the contingent environmental conditions is the key feature to such a disorder. The H/PTSD-Spectrum, therefore, should be seen as a syndrome caused by an acquired inability (increased salience) concerning everyday daily life events.

## Figures and Tables

**Table 1:** Bivariate correlation between the severity of stress sensitivity and other clinical aspects (only significant correlations were reported).

	Spearman’s Rho	Sig. (2-Tailed)
Demographic data		
Income	0.41	0.028
DAH-Q		
II. Mental Status areas altered	0.46	0.005
III. Legal problems	0.39	0.029
VIIa. Lifetime Different treatments	0.35	0.041
VIIb. Current treatment load	0.36	0.033
Psychopathology		0.000
SCL90 Global Score Index	0.63	0.000
SCL90 Positive symptom Index	0.60	0.002
SCL90 Positive symptom distress (severity)	0.45	0.000
F1. Worthlessness/Being trapped	0.58	0.000
F2. Somatic Symptoms	0.60	0.000
F3. Sensitivity/Psychoticism	0.57	0.000
F4. Panic/Anxiety	0.49	0.000
F5. Violence/Suicide	0.45	0.000
Deltito Wellness Scale		
Best week (last five years) contrast	−0.36	0.018

**Table 2:** Differences between patients with and without H/PTSD-S according to the nonparametric test.

	Stat	With H/PTSD-S N = 34	Without H/PTSD-S N = 10		
Demographics					*p*
Female sex	N (%)	11 (32.4%)	0 (0.0%)		0.046
Income, poor	N (%)	11 (32.4%)	0 (0.0%)		0.046
DAH-Q					
F3. Occupation, present	N (%)	9 (26.5%)	8 (80.0%)		0.055
F7. Legal problems, present	N (%)	19 (55.9%)	1 (10.0%)		0.045
				U *	*p*
F2. Mental status areas altered	Median	0.42	0.20	50.50	0.022
SCL-90 Data					
SCL90 Global score index	Median	1.22	0.40	52.00	0.001
SCL90 Positive symptom index	Median	61.50	27.5	53.00	0.001
SCL90 Positive symptom distress (severity)	Median	1.89	1.55	94.00	0.012
F1. Worthlessness/Being Trapped	Median	1.66	0.47	65.00	0.002
F2. Somatic Symptoms	Median	1.36	0.44	92.00	0.000
F3. Sensitivity/Psychoticism	Median	0.94	0.20	69.00	0.004
F4. Panic Anxiety	Median	0.66	0.05	72.00	0.005
F5. Violence/Suicide	Median	1.25	0.50	81.00	0.012
DSWS Data					
Poor week (last year) contrast	Median	5.50	19.50	242.00	0.022
Best week (last five years) contrast	Median	16.50	27.00	219.00	0.006

* Mann–Whitney U test.

## Data Availability

At the researchers’ location.
